# Sex-specific effects of implementing a high-sensitivity troponin I assay in patients with suspected acute coronary syndrome: results from SWEDEHEART registry

**DOI:** 10.1038/s41598-020-72204-2

**Published:** 2020-09-17

**Authors:** Dorien M. Kimenai, Bertil Lindahl, Tomas Jernberg, Otto Bekers, Steven J. R. Meex, Kai M. Eggers

**Affiliations:** 1 Central Diagnostic Laboratory, Maastricht University Medical Center, Post Office Box 5800, 6202 AZ Maastricht, The Netherlands; 2 CARIM School for Cardiovascular Diseases, Maastricht University, Maastricht, The Netherlands; 3 Department of Medical Sciences, Cardiology, Uppsala University, Uppsala, Sweden; 4 Uppsala Clinical Research Center, Uppsala University, Uppsala, Sweden; 5 Department of Clinical Sciences, Danderyd University Hospital, Karolinska Institutet, Stockholm, Sweden

**Keywords:** Diagnostic markers, Acute coronary syndromes

## Abstract

Using high-sensitivity cardiac troponin (hs-cTn) assays with sex-specific 99th percentiles may improve management of patients with suspected acute myocardial infarction (AMI). We investigated the impact of transitioning from a conventional troponin I assay to a high-sensitivity assay with sex-specific thresholds, in patients with suspected acute coronary syndrome admitted to Swedish coronary care units. Based on data from SWEDEHEART registry (females, n = 4,819/males, n = 7,670), we compared periods before and after implementation of hs-cTnI assay (Abbott) using sex-specific 99th percentiles. We investigated differences on discharge diagnosis, in-hospital examinations, treatments, and clinical outcome. Upon implementation of the hs-cTnI assay, proportion of patients with troponin levels above diagnostic AMI threshold increased in women and men by 24.3% versus 14.8%, respectively. Similarly, incidence of AMI increased by 11.5% and 9.8%. Diagnostic interventions and treatments increased regardless of sex. However, these associations did not persist following multivariable adjustment, probably due to the effect of temporal management trends during the observation period. Overall, no risk reduction on major adverse cardiovascular events was observed (HR: 0.91 [95% CI 0.80–1.03], *P* = 0.126). The implementation of hs-cTnI assay together with sex-specific 99th percentiles was associated with an increase in incidence of AMI regardless of sex, but had no major impact on clinical management and prognosis.

## Introduction

Over the years, the improved sensitivity of cardiac troponin assays has allowed shorter time intervals between serial cardiac troponin measurements, from 6–9 to ≤ 3 h to establish diagnosis of AMI^1–3^. Nowadays, these high-sensitivity cardiac troponin (hs-cTn) assays are recommended over conventional cardiac troponin (cTn) assays for AMI diagnosis^4,5^. The 99th percentiles of hs-cTn derived from a healthy reference population are the recommended threshold for AMI diagnosis. Although the use of 99th percentiles had been advocated already in 2000 ESC/ACC guidelines, this had not been feasible until the introduction of hs-cTn assays^6^. Overall, implementation of high-sensitivity cardiac troponin I (hs-cTnI) and T (hs-cTnT) assays revealed a higher proportion of patients being reclassified for myocardial injury and eligible for beneficial therapies^7,8^. Awareness has been raised that women are underdiagnosed for AMI and may receive less evidence-based treatment^9–11^. As female-specific 99th percentiles for troponins are lower than male-specific 99th percentiles, it is conceivable that implementation of a high-sensitivity assay with sex specific diagnostic thresholds will reduce underdiagnosis of AMI in women^12,13^. Studies that focus on the sex-specific clinical effect of implementing hs-cTn assays using sex-specific thresholds are scarce, and there is in particular limited data available from real-life studies^9,11,14^. During the changeover from cTn assays to hs-cTn assays, the Abbott assay was the only hs-cTnI method used in Sweden until the end of 2018. All Swedish hospitals who implemented hs-cTnI assay of Abbott, also applied sex-specific 99th percentiles for AMI diagnosis. The aims with the present study were therefore to (1) investigate differences between cTn period and hs-cTn period on proportion of AMI diagnosis in women and men, (2) assess the effect of implementation of hs-cTnI assay on in-hospital examinations and treatments in women and men, and (3) investigate whether this affected outcome in major adverse cardiac events and all-cause mortality as compared with cTn assays.

## Results

### Study population

A total of 15,313 patients were admitted during respective observation periods at the nine hospitals (Supplementary Table S1), and after exclusion of 2,824 patients that did not meet the inclusion criteria, 12,489 patients were available for analysis (cTn period, n = 6,385; hs-cTnI, n = 6,104, Supplementary Fig. S1). The proportion of women was in both periods 39%. Baseline characteristics and in-hospital treatment per study period, stratified by sex, are presented in Table 1 (patients with troponin levels above the respective AMI threshold, Supplementary Table S2, patients with troponin levels below the respective AMI threshold, Supplementary Table S3). In both periods, women are older with a median age difference of four years. Overall, hs-cTnI period included older patients and a higher proportion of individuals with hypertension, diabetes and hyperlipidaemia. Number of patients with ST-segment depression was higher in hs-cTnI period than in cTn period.

**Table 1:** Baseline characteristics and in-hospital treatment per study period and stratified by sex.

	Overall			Women			Men		
	cTn period (n = 6,385)	hs-cTnI period (n = 6,104)	*P* value	cTn period (n = 2,467)	hs-cTnI period (n = 2,352)	*P* value	cTn period (n = 3,918)	hs-cTnI period (n = 3,752)	*P* value
Age (years)	71 (62–80)	72 (64–80)	< 0.001	74 (64–83)	75 (67–83)	0.005	70 (61–78)	71 (62–78)	0.025
Female sex	2,467 (38.6%)	2,352 (38.5%)	0.904	–	–		–	–	
**Risk factors**									
Current smoking	867 (13.6%)	800 (13.1%)	0.410	293 (11.9%)	314 (13.4%)	0.127	574 (14.7%)	486 (13.0%)	0.028
Hypertension	3,187 (50.0%)	3,786 (62.0%)	< 0.001	1,341 (54.4%)	1,541 (65.5%)	< 0.001	1,846 (47.2%)	2,245 (59.9%)	< 0.001
Diabetes	1,544 (24.2%)	1,655 (27.1%)	< 0.001	582 (23.6%)	623 (26.5%)	0.020	962 (24.6%)	1,032 (27.5%)	0.003
Hyperlipidemia	2,718 (42.6%)	2,800 (45.9%)	< 0.001	916 (37.1%)	974 (41.4%)	0.002	1,802 (46.0%)	1,826 (48.7%)	0.019
Body mass index (kg/m^2^)	27 (24–30)	27 (24–30)	0.395	27 (24–30)	27 (24–30)	0.272	27 (25–30)	27 (25–30)	0.847
eGFR (CKD-EPI, mL/kg/m^2^)	74 (53–89)	76 (56–90)	< 0.001	70 (49–86)	71 (51–87)	0.024	76 (56–90)	79 (61–91)	< 0.001
**History**									
Previous AMI	2,385 (37.4%)	2,233 (36.6%)	0.369	811 (32.9%)	776 (33.0%)	0.929	1,574 (40.2%)	1,457 (38.8%)	0.226
Previous PCI/CABG	2,049 (32.1%)	2,030 (33.3%)	0.166	588 (23.9%)	595 (25.3%)	0.241	1,461 (37.3%)	1,435 (38.3%)	0.387
Heart failure	934 (14.6%)	777 (12.7%)	0.002	326 (13.2%)	250 (10.6%)	0.006	608 (15.5%)	527 (14.1%)	0.069
Previous stroke	604 (9.5%)	531 (8.7%)	0.140	225 (9.1%)	205 (8.7%)	0.623	379 (9.7%)	326 (8.7%)	0.136
COPD	493 (7.7%)	460 (7.5%)	0.711	206 (8.4%)	222 (9.4%)	0.188	287 (7.3%)	238 (6.3%)	0.094
Dementia	32 (0.5%)	12 (0.2%)	0.004	10 (0.4%)	2 (0.5%)	0.303	22 (0.6%)	7 (0.2%)	0.008
Previous/present cancer	312 (4.9%)	238 (3.9%)	0.008	76 (3.1%)	70 (3.0%)	0.867	236 (6.0%)	168 (4.5%)	0.003
**ECG findings**									
Sinus rhythm	5,153 (82.8%)	5,102 (83.9%)	0.112	2,011 (83.8%)	1,982 (84.5%)	0.492	3,142 (82.2%)	3,140 (83.4%)	0.140
Atrial fibrillation/flutter	881 (14.2%)	785 (12.9%)	0.042	338 (14.1%)	302 (12.9%)	0.224	543 (14.2%)	483 (12.9%)	0.104
ST-segment depression	1,227 (19.7%)	1,413 (23.2%)	< 0.001	521 (21.7%)	572 (24.4%)	0.029	706 (18.5%)	841 (22.5%)	< 0.001
T-wave inversion	616 (9.9%)	563 (9.3%)	0.222	272 (11.3%)	230 (9.8%)	0.086	344 (9.0%)	333 (8.9%)	0.889
Other ST segment changes	1,181 (19.0%)	1,211 (19.9%)	0.197	411 (17.1%)	444 (18.9%)	0.108	770 (20.1%)	767 (20.5%)	0.688
No ST-segment changes	3,197 (51.4%)	2,897 (47.6%)	< 0.001	1,194 (49.8%)	1,099 (46.9%)	0.044	2,003 (52.4%)	1,798 (48.1%)	< 0.001
**In-hospital examinations and interventions**									
Echocardiography	2,559 (40.1%)	3,336 (54.7%)	< 0.001	901 (36.5%)	1,270 (54.0%)	< 0.001	1,658 (42.3%)	2,066 (55.1%)	< 0.001
Coronary angiography	2,886 (45.2%)	3,816 (62.5%)	< 0.001	945 (38.3%)	1,311 (55.7%)	< 0.001	1,941 (49.5%)	2,505 (66.8%)	< 0.001
PCI	1,542 (24.2%)	2,031 (33.3%)	< 0.001	432 (17.5%)	557 (23.7%)	< 0.001	1,110 (28.3%)	1,474 (29.3%)	< 0.001
CABG	78 (1.3%)	130 (2.1%)	0.001	18 (0.8%)	27 (1.1%)	0.199	60 (1.6%)	103 (2.7%)	0.001
**Coronary status**^a^									
Non-conclusive	8 (0.3%)	1 (0.0%)	0.006	4 (0.4%)	0 (0.0%)	0.020	4 (0.2%)	1 (0.0%)	0.104
Normal/atheromatosis	825 (28.6%)	1,173 (31.3%)	0.019	368 (38.2%)	571 (43.8%)	0.008	457 (23.8%)	602 (24.6%)	0.539
1–2 vessel disease	1,531 (53.1%)	1,887 (50.3%)	0.024	458 (47.6%)	565 (43.3%)	0.045	1,073 (55.9%)	1,322 (54.0%)	0.222
3 vessel disease/left main	520 (18.0%)	691 (18.4%)	0.686	133 (13.8%)	168 (12.9%)	0.520	387 (20.1%)	523 (21.4%)	0.325
**Left ventricular ejection fraction**^b^									
≥ 50%	1,630 (64.5%)	2,288 (69.0%)	< 0.001	605 (68.1%)	890 (70.7%)	0.215	1,025 (62.5%)	1,398 (68.0%)	< 0.001
31–49%	664 (26.3%)	780 (23.5%)	0.017	216 (24.3%)	282 (22.4%)	0.300	448 (27.3%)	498 (24.2%)	0.034
≤ 30%%	233 (9.2%)	246 (7.4%)	0.014	67 (7.5%)	87 (6.9%)	0.611	166 (10.1%)	159 (7.7%)	0.012
Duration of hospital stays (days)	2 (1–4)	3 (2–4)	< 0.001	2 (1–4)	3 (2–5)	< 0.001	3 (1–4)	3 (2–4)	< 0.001
**Medication at discharge**^c^									
Aspririn	4,158 (68.2%)	4,088 (69.2%)	0.246	1,455 (61.6%)	1,478 (65.0%)	0.018	2,703 (72.4%)	2,610 (71.8%)	0.603
P2Y12 inhibitors	2,782 (45.6%)	3,277 (55.5%)	< 0.001	919 (38.9%)	1,109 (48.7%)	< 0.001	1,863 (49.9%)	21,768 (59.7%)	< 0.001
Anticoagulants	807 (13.2%)	561 (9.5%)	< 0.001	302 (12.8%)	202 (8.9%)	< 0.001	505 (13.5%)	359 (9.9%)	< 0.001
Β-blockers	4,303 (70.6%)	4,374 (74.0%)	< 0.001	1,614 (68.3%)	1,658 (72.9%)	0.001	2,689 (72.0%)	2,716 (74.7%)	0.008
ACEI/ARB	3,740 (61.4%)	4,159 (70.4%)	< 0.001	1,343 (56.9%)	1,574 (69.2%)	< 0.001	2,397 (64.2%)	2,585 (71.1%)	< 0.001
Statins	4,129 (67.7%)	4,470 (75.6%)	< 0.001	1,420 (60.1%)	1,570 (69.0%)	< 0.001	2,709 (72.5%)	2,900 (79.8%)	< 0.001

*ACEI* angiotensin-converting-enzyme inhibitor, *AMI* acute myocardial infarction, *ARB* angiotensin II receptor blockers, *CABG* coronary artery bypass graft, *CKD-EPI* chronic kidney disease epidemiology collaboration equation, *COPD* chronic obstructive pulmonary disease, *eGFR* estimated glomerular filtration rate, *PCI* percutaneous coronary intervention.

^a^n = 6,636, ^b^n = 5,841, ^c^assessed in in-hospital survivors (n = 12,233).

### Discharge diagnoses per study period

**Figure 1:**
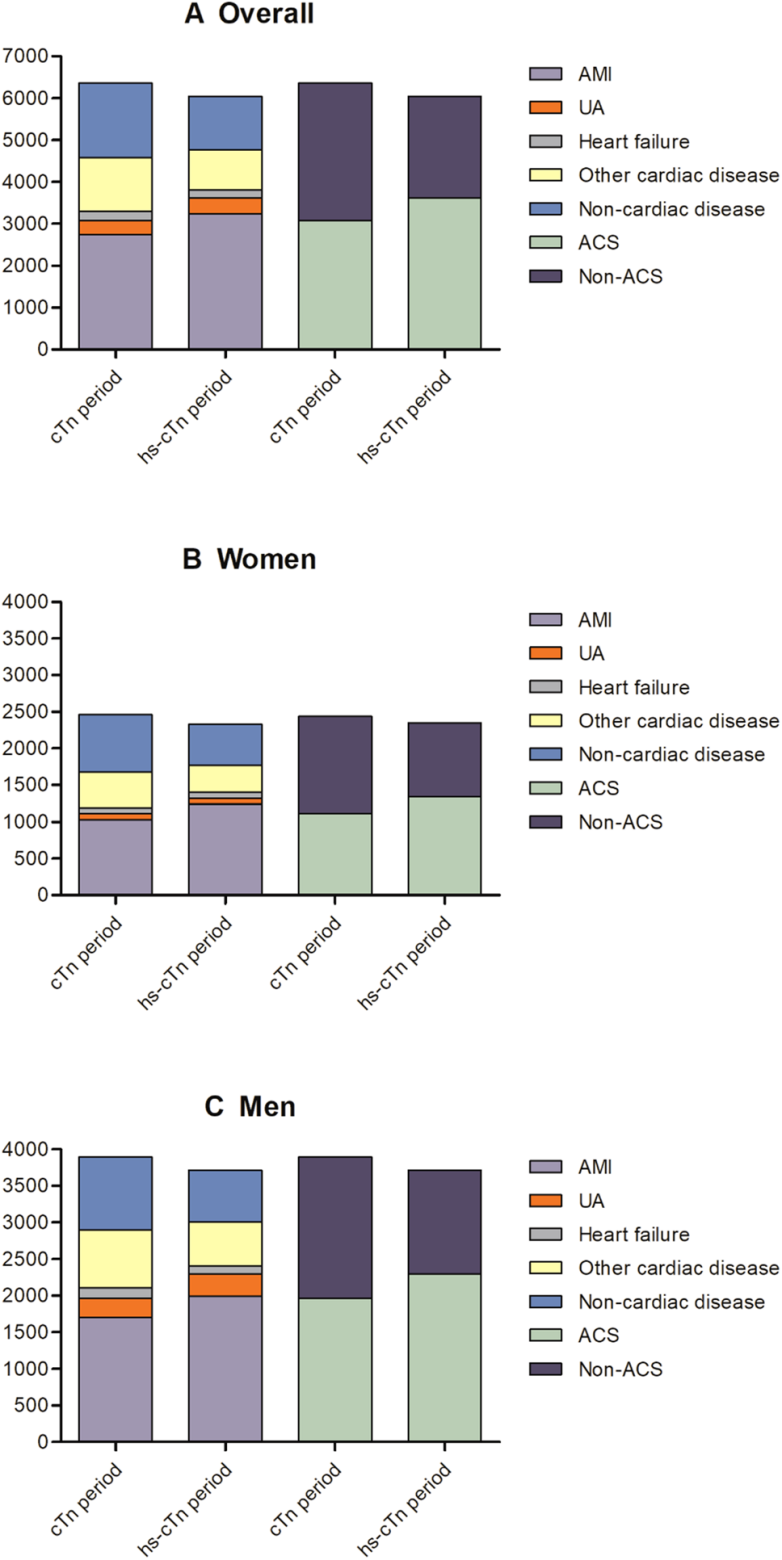
Discharge diagnoses per study period in all patients (**A**), women (**B**) and men (**C**).

The number of patients with a troponin level above AMI threshold increased in hs-cTnI period (cTn period, 3,482/6,354 versus hs-cTnI period, 4,426/6,044; from 54.8 to 73.2%). Stratified by sex, the proportion of females and males with troponin levels above the AMI threshold increased in hs-cTnI period by 24.3% and 14.9% as compared with cTn period, respectively (women, from 53.1 to 77.4%; men, from 55.8 to 70.7%). Proportion of patients with troponin levels below AMI threshold decreased significantly with 18.4% in the hs-cTnI period (cTn period, 2,872/6,354 versus hs-cTnI period, 1,618/6,044; from 45.2 to 26.8%). Overall, proportion of patients diagnosed with ACS increased with 11.4% in hs-cTnI period as compared with cTn period (Fig. 1, Table 2). This shift was particularly the result of a higher proportion in discharge diagnosis of AMI (cTn period, 2,736/6,345 versus hs-cTnI period, 3,235/6,044, from 43.1 to 53.5%). Women and men showed a similar pattern with increased discharge diagnosis of AMI for hs-cTnI period as compared with cTn period (11.5% and 9.8% increase in women and men, respectively). Less patients received the diagnosis of non-cardiac disease in hs-cTnI period, and this observation was similar for both women and men.

**Table 2:** Discharge diagnoses per study period, stratified by sex.

Overall^a^	cTn period	hs-cTnI period	Absolute change (%)	*P* value
	(n = 6,354)	(n = 6,044)		
**ACS**	3,079 (48.5%)	3,618 (59.9%)	+ 11.4	< 0.001
Myocardial infarction	2,736 (43.1%)	3,235 (53.5%)	+ 10.4	< 0.001
Unstable angina	343 (5.4%)	383 (6.3%)	+ 0.9	0.026
**Non-ACS**	3,275 (51.5%)	2,426 (40.1%)	− 11.4	< 0.001
Heart failure	219 (3.4%)	185 (3.1%)	− 0.3	0.227
Other cardiac disease	1,275 (20.1%)	966 (16.0%)	− 4.1	< 0.001
Non-cardiac disease	1,781 (28.0%)	1,275 (21.1%)	− 6.9	< 0.001

Women	(n = 2,458)	(n = 2,328)	Absolute change (%)	*P* value
**ACS**	1,116 (45.4%)	1,323 (56.8%)	+ 11.4	< 0.001
Myocardial infarction	1,030 (41.9%)	1,242 (53.4%)	+ 11.5	< 0.001
Unstable angina	86 (3.5%)	81 (3.5%)	–	0.971
**Non-ACS**	1,342 (54.6%)	1,005 (43.2%)	− 11.4	< 0.001
Heart failure	76 (3.1%)	74 (3.2%)	+ 0.1	0.863
Other cardiac disease	486 (19.8%)	372 (16.0%)	− 3.8	0.001
Non-cardiac disease	780 (31.7%)	559 (24.0%)	− 7.7	< 0.001

Men	(n = 3,896)	(n = 3,716)	Absolute change (%)	*P* value
**ACS**	1,963 (50.4%)	2,295 (61.8%)	+ 11.4	< 0.001
Myocardial infarction	1,706 (43.8%)	1,993 (53.6%)	+ 9.8	< 0.001
Unstable angina	257 (6.6%)	302 (8.1%)	+ 1.5	0.011
**Non-ACS**	1,933 (49.6%)	1,421 (38.2%)	− 11.4	< 0.001
Heart failure	143 (3.7%)	111 (3.0%)	− 0.7	0.097
Other cardiac disease	789 (20.3%)	594 (16.0%)	− 4.3	< 0.001
Non-cardiac disease	1,001 (25.7%)	716 (19.3%)	− 6.4	< 0.001

*ACS* acute coronary syndrome, *cTn* conventional cardiac troponin, *hs-cTnI* high-sensitivity cardiac troponin I.

^a^Missing values: cTn period, n = 31 (M, n = 22; F, n = 9), hs-cTnI period, n = 60 (M, n = 36; F, n = 24).

### In-hospital examinations and treatment

Tables 1 and 3 provide detailed information on management changes following transition from conventional to hs-cTnI assay. Proportion of individuals that received in-hospital echocardiography was higher during hs-cTnI period as compared with cTn period regardless of hs-cTnI status (Supplementary Tables S2 and S3). Greatest numerical increases were noted among women (women, from 36.5 to 54.0%; men, from 42.5 to 55.1%), in particular those who had troponin levels below the AMI threshold (women, from 18.3 to 31.9%; men, from 22.3 to 32.6%). Proportions of women and men found to have normal left ventricular ejection fraction (i.e. ≥ 50%) increased by 2.6% and 5.5%, respectively, from cTn period to hs-cTnI period. Use of coronary angiography increased in both sexes, regardless of hs-cTnI-status, although proportion of individuals that underwent coronary angiography remained lower for women than men in hs-cTnI period (56% versus 67%). Greatest numerical increase on coronary angiography was seen in men with troponin levels below the AMI threshold (women, from 23.2 to 37.6%; men, from 28.6 to 50.8%). Proportions of patients found to have normal coronaries or non-occlusive atheromatosis increased by 5.6% (*P* = 0.008) among women and 0.8% (*P* = 0.539) among men. Increased use of coronary angiography more often resulted in percutaneous coronary invention (PCI)/coronary artery bypass graft (CABG), particularly in men, regardless of hs-cTnI-status. The prescription of medications increased in the hs-cTnI period, with the exception of prescription of antiplatelets that decreased in the hs-cTnI period (women, from 12.8 to 8.9%; men, from 13.5 to 9.9%).

**Table 3:** In-hospital examinations and treatments in the total population and in patients with troponin levels above and below the respective AMI threshold.

	Total population (women, n = 4,684; men, n = 7,499)			Patients with troponin levels above the respective AMI threshold (women, n = 3,059; men, n = 4,733)			Patients with troponin levels below the respective AMI threshold (women, n = 1,625; men, n = 2,766)		
	OR (95% CI)	*P* value	P_int_	OR (95% CI)	*P* value	P_int_	OR (95% CI)	*P* value	P_int_
**All**									
UFH, LMWH, or fondaparinux	0.98 (0.87–1.10)	0.711	0.034	0.95 (0.82–1.10)	0.467	0.416	0.62 (0.45–0.85)	0.003	0.823
Echocardiography	0.94 (0.84–1.06)	0.321	0.004	0.82 (0.71–0.95)	0.006	0.442	1.06 (0.84–1.35)	0.625	0.113
Coronary angiography	0.99 (0.88–1.11)	0.822	0.805	1.05 (0.91–1.21)	0.523	0.512	0.68 (0.54–0.86)	0.001	0.150
PCI/CABG	0.92 (0.82–1.03)	0.156	0.426	0.93 (0.81–1.06)	0.285	0.261	0.71 (0.56–0.91)	0.007	0.082
Antiplatelets at discharge^a^	0.90 (0.79–1.03)	0.132	0.256	0.76 (0.63–0.92)	0.004	0.994	0.96 (0.76–1.19)	0.697	0.323
ACEI/ARB at discharge^a^	0.94 (0.83–1.06)	0.296	0.006	0.87 (0.75–1.02)	0.086	0.039	0.98 (0.79–1.22)	0.882	0.885
Statins at discharge	1.02 (0.90–1.16)	0.740	0.870	0.90 (0.76–1.07)	0.238	0.403	1.14 (0.92–1.42)	0.225	0.800
Length of stay > 3 days	1.05 (0.94–1.19)	0.387	0.074	1.02 (0.89–1.17)	0.802	0.791	0.71 (0.53–0.96)	0.025	0.225
**Women**									
UFH, LMWH, or fondaparinux	1.10 (0.91–1.33)	0.320	–	0.94 (0.75–1.17)	0.566	–	0.56 (0.31–0.98)	0.043	–
Echocardiography	1.08 (0.90–1.30)	0.429	–	0.86 (0.69–1.08)	0.189	–	1.30 (0.96–1.96)	0.208	–
Coronary angiography	0.98 (0.81–1.19)	0.838	–	1.01 (0.80–1.26)	0.955	–	0.59 (0.40–0.88)	0.009	–
PCI/CABG	0.82 (0.67–1.00)	0.047	–	0.80 (0.64–1.00)	0.052	–	0.48 (0.30–0.79)	0.004	–
Antiplatelets at discharge^a^	0.95 (0.78–1.16)	0.623	–	0.76 (0.58–1.01)	0.055	–	0.85 (0.60–1.21)	0.367	–
ACEI/ARB at discharge^a^	1.15 (0.95–1.40)	0.151	–	1.08 (0.35–1.37)	0.545	–	1.04 (0.73–1.49)	0.818	–
Statins at discharge^a^	1.05 (0.86–1.27)	0.651	–	0.86 (0.68–1.10)	0.239	–	1.18 (0.83–1.68)	0.370	–
Length of stay > 3 days	1.12 (0.92–1.35)	0.254	–	0.97 (0.78–1.21)	0.782	–	0.60 (0.36–1.00)	0.052	–
**Men**									
UFH, LMWH, or fondaparinux	0.91 (0.79–1.06)	0.220	–	0.97 (0.81–1.17)	0.766	–	0.63 (0.43–0.92)	0.015	–
Echocardiography	0.87 (0.75–1.01)	0.063	–	0.81 (0.68–0.97)	0.024	–	0.95 (0.71–1.27)	0.716	–
Coronary angiography	1.10 (0.86–1.17)	0.951	–	1.15 (0.95–1.40)	0.158	–	0.70 (0.52–0.93)	0.015	–
PCI/CABG	1.00 (0.86–1.16)	0.982	–	1.09 (0.91–1.30)	0.354	–	0.76 (0.57–1.02)	0.069	–
Antiplatelets at discharge^a^	0.88 (0.73–1.05)	0.153	–	0.78 (0.60–1.02)	0.068	–	0.98 (0.75–1.29)	0.893	–
ACEI/ARB at discharge^a^	0.82 (0.70–0.96)	0.014	–	0.76 (0.62–0.93)	0.008	–	0.93 (0.71–1.22)	0.588	–
Statins at discharge^a^	1.02 (0.86–1.22)	0.811	–	1.00 (0.79–1.26)	0.991	–	1.07 (0.81–1.42)	0.623	–
Length of stay > 3 days	1.01 (0.87–1.18)	0.914	–	1.03 (0.87–1.23)	0.730	–	0.77 (0.53–1.10)	0.147	–

*ACEI* angiotensin-converting-enzyme inhibitor, *ARB* angiotensin II receptor blockers, *CABG* coronary artery bypass graft, *COPD* chronic obstructive pulmonary disease; *LMWH* low-molecular-weight heparin, *PCI* percutaneous coronary intervention, *UFH* unfractionated heparin. Reference category: cTn period.

^a^Data from hospital survivors, women, n = 4,590; men, n = 7,346. The table presents the associations of hs-cTnI period (as compared with cTn period, reference category) with in-hospital examinations and treatments, stratified by sex and by patients with troponin levels above and below the respective AMI threshold. The in-hospital examinations and treatments were taken as dependent variable in the model. Adjustment was made for sex (if appropriate), year of admission and hospital site. Data are given as OR with 95% CI. P_interaction_ refers to the interaction term of the used cTn assay (conventional or high-sensitivity) on the association of sex with the respective examination or treatment (cTn assay * sex).

In analyses adjusted for hospital site and admission year, the overall difference between in-hospital examinations and treatments between the cTn period and hs-cTnI period was attenuated (Table 3). Nonetheless, there were significant reductions in the use of intravenous/subcutaneous anticoagulants, coronary angiography and revascularization procedures in patients with cTn levels below the AMI threshold, and the latter was mainly apparent in women (P_interaction_ = 0.082). Significant interactions between cTn period and sex were observed for use of intravenous/subcutaneous anticoagulants, echocardiography and prescription of angiotensin-converting enzyme inhibitors (ACEI)/angiotensin-II receptor blockers (ARB) (P_interaction_ = 0.034; P_interaction_ = 0.004; P_interaction_ = 0.006, respectively). In general, the use of these measures tended to increase in women and to decrease in men. In particular more women with troponin levels above the AMI threshold were discharged with ACEI/ARB in the hs-cTnI period as compared to men who had troponin levels above the AMI threshold in the same period (P_interaction_ = 0.039).

### Outcome

After exclusion of readmitted patients, 10,226 patients were included for analysis. Information on major adverse cardiovascular events (MACE) was collected during 22,083 patient years (cTn period: 17,501 patient years; hs-cTnI period: 4,582 patient years) and on all-cause mortality during 27,876 patient years (cTn period: 21,338 patient years; hs-cTnI period: 6,538 patient years). Incidence rates tended to be higher in hs-cTnI period regardless of sex (cTn period: 6.6/100 patient-years; hs-cTnI period: 8.9/100 patient-years).

After adjustment, no overall difference in MACE and all-cause mortality was noted in hs-cTnI group as compared with cTn group, respectively (HR: 0.91 [95% CI 0.80–1.03]; HR: 0.87 [95% CI 0.75–1.00]). For patients with troponin levels above the respective AMI threshold, no reduction in risk on MACE and all-cause mortality was noted after adjustment, without difference between sexes (MACE, P_interaction_ = 0.576; all-cause mortality, P_interaction_ = 0.210, Supplementary Table S4). In men with troponin levels below the AMI threshold, significant reductions in risk on MACE and all-cause mortality were noted in the hs-cTnI period (HR: 0.61 [95% CI 0.40–0.92]; HR: 0.47 [95% CI 0.27–0.82]). Conversely, no major changes in risk on MACE and all-cause mortality were observed in women with troponin levels below the AMI threshold between cTn period and hs-cTn period (HR: 0.79 [95% CI 0.45–1.40]; HR: 0.73 [95% CI 0.38–1.41]).

## Discussion

In this real-life study, we investigated changes in case mix of women and men with suspected ACS admitted to CCU, their clinical management and outcome following transition from a conventional to a hs-cTnI assay used together with sex-specific thresholds.

Our study has three main findings. First, implementation of hs-cTnI assay using sex-specific thresholds was associated with an increase in admission of patients with troponin levels above diagnostic AMI threshold and those at increased risk, i.e. patients with higher age, cardiovascular risk factors or ischemic ECG changes. This is in line with previous studies^8,11,15–17^. Similarly, more patients received the diagnosis of AMI and less patients received the diagnosis of non-cardiac disease in hs-cTnI period. The increase in proportion patients with troponin levels above the AMI threshold was higher for women than men (absolute increase: women, 24.8% versus men, 14.8%). This is expected since female-specific threshold in hs-cTnI period relatively decreased compared to overall threshold used in cTn-period^11^. As a consequence, incidence of AMI among women increased by 11.5%, while the incidence of AMI in men increased by 9.8%.

Second, in-hospital examinations and treatments increased in hs-cTnI period as compared with cTn period. However, our adjusted analysis indicate that the divergence between cTn and hs-cTnI period seems particularly explained by temporal management trends irrespective of the implementation of hs-cTnI assay.

Third, no overall difference in outcome was noted after changeover to hs-cTnI assay. This is in line with results of High-STEACS study and not unexpected since management changes in patients with cTn levels around the 99th percentile usually only have limited effect on hard outcomes^7,11^. For example, invasive assessment does not reduce risk unless hs-cTnT levels are three times above the 99th percentile^18^. This supports the hypothesis that 99th percentiles might not be the best benchmark to manage patients with suspected AMI^7^. Interestingly however, lower risk estimates were noted in patients with troponin levels below the AMI threshold. This indicates that true low-risk patients are identified more reliably by the hs-cTnI assay.

The implementation of this assay was accompanied both with introduction of sex-specific thresholds and lowering of diagnostic threshold to 99th percentile at some hospitals. Accordingly, it is difficult to disentangle to which degree any of these components may have contributed to changes seen in our analysis. However, some assumptions are close at hand. The changeover to hs-cTnI assay was associated with an increase of at-risk patients being admitted to CCU, similar as seen in other investigations^8^. Nonetheless, no overall increase in diagnostic examinations and treatment was noted in adjusted analyses. This indicates that changes over time^19^ to a greater degree affected patient management than the implementation of the hs-cTnI assay in itself. However, patients with hs-cTnI levels below the AMI threshold had apparently more often been perceived as having low risk, as reflected by significant decreases in the use of intravenous/subcutaneous anticoagulants, coronary angiography and revascularization procedures. At the same, the increase in the number of patients with hs-cTnI levels above the AMI threshold was not associated with an inappropriate inflation in the use of diagnostic examinations and treatments.

The use of sex-specific 99th percentiles appeared to affect management of women and men in several, partly unexpected aspects. Conceptually, lower female-specific 99th percentile would be expected to increase sensitivity while lowering specificity. This may have contributed to the increased prescription of ACEI/ARB in women. At the same time, the prescription of ACEI/ARB decreased in men, intriguingly in particular among those with troponin levels above the AMI threshold. This was accompanied by corresponding changes in the use of echocardiography, a finding that we are unable to explain on the basis of the present data. Clearly, further investigation is needed.

What are the clinical implications of our study? The implementation of the hs-cTnI assay increased the proportion of AMI diagnosis significantly by approximately 10% in patients presenting at the CCU with suspected acute coronary syndrome, and this was similar for women and men. We only noted small changes in diagnostic examinations and treatment, mostly in women and men with troponin levels below the AMI threshold. These estimates however, should be acknowledged as rather conservative estimates given the possibility of collinearity between admission year and study period in the multivariable analyses. We feel that it is now of great importance to unravel what the most optimized ‘rule-in/rule-out’ algorithm is for hs-cTn assays to improve treatment and outcome in patients with suspected acute coronary syndrome. Intriguingly, we also noted that the proportion of women who presented at the CCU with suspected acute coronary syndrome did not change over time and was in both periods 39%, despite the use of sex-specific thresholds. This observation may suggest sex referral bias at cardiac emergency unit and requires further urgent attention.

A number of limitations of this study should be mentioned. (1) Only hs-cTnI assay from Abbott is studied, and we cannot make firm conclusions on sex-specific impact of other hs-cTn assays. (2) The design of our study is observational, and our results reflect combined effects of changing to a high-sensitivity assay and using sex-specific thresholds. It is difficult to separate which aspect in assay change might have had greatest impact on the changes found in our analysis. Accordingly, inferences on causality should be made with caution. Moreover, residual confounding may persist. (3) Patients with troponin levels above the AMI threshold determined with high-sensitivity assay are enriched with less sick patients that might have remained undetected when using conventional cTn assay. This introduces selection bias which could have affected our results. (4) We investigated patients with suspected ACS admitted at CCU. Accordingly, we are unable to comment to which extent men at risk but being “troponin-negative” by means of the relatively higher male-specific 99th percentile might not have been regarded being in need of CCU admission. This implies a residual risk of underdiagnosis and undertreatment in these individuals. In addition, generalization of our findings to emergency department patients should be done with caution. (5) The lower proportion of women as compared with men admitted to CCU may suggest sex referral bias at cardiac emergency unit. This is in line with recent data, showing a systematic sex-bias in the early management of patients with suspected ACS^20^. Accordingly, our results probably reflect the minimum effect on differences in diagnosis, management and outcome between women and men with suspected ACS admitted at CCU. (6) In the SWEDEHEART registry, only peak troponin levels are registered. We were therefore not able to investigate sex-differences regarding the relation between temporal cTn changes and in-hospital examinations and treatments. Greater temporal hs-cTn changes remaining below the AMI-threshold may for example, have led to more invasive diagnostic procedures in men compared to women. This hypothesis needs to be assessed in future research. (7) As the SWEDEHEART registry is based on real-world data, no standardized “rule-in/rule-out” algorithm for diagnosis of AMI was applied in hospitals. However, all hospitals introduced sex-specific thresholds during hs-cTnI period, and we were therefore able to investigate the impact of implementing a hs-cTnI assay using sex-specific thresholds in clinical practice.

In conclusion, implementation of a hs-cTnI assay using sex-specific thresholds was associated with an increase in the proportion of women and men with troponin levels above diagnostic AMI threshold, and has led to more AMI diagnosis at discharge. The increase of in-hospital examinations and treatments after hs-cTnI implementation seems particularly explained by temporal management trends irrespective of the implementation of hs-cTnI assay. Overall, implementation of hs-cTnI assay using sex-specific 99th percentiles had only limited effect on clinical outcome in both women and men.

## Methods

### Study population

We used data from Swedish Web based system for Enhancement and Development of Evidence based care in Heart disease Evaluated According to Recommended Therapies (SWEDEHEART) registry recorded between time period from August, 2010 till the end of May, 2018. The SWEDEHEART registry includes all individuals with suspected ACS admitting at a CCU or other specialized facility at all Swedish acute cardiac care hospitals^8^. Patients included in the registry are informed about their participation which they have the right to decline. Written consent is not required according to Swedish law. In this study, only hospitals who implemented Abbott ARCHITECT STAT cardiac troponin I assay are included (n = 9). Based on the use of hs-cTnI assay, patients were divided into those admitted before implementation of hs-cTnI assay and those admitted thereafter. Patients diagnosed with ST-segment elevation myocardial infarction and those who lacked a troponin measurement were excluded from analyses. The present manuscript follows the Strengthening the Reporting of Observational Studies in Epidemiology guidelines^21^. The study was conducted according to principles of 1975 Declaration of Helsinki and approved by Regional Ethical Review Board in Stockholm (2012/60-31/2).

### Cardiac troponin measurements

The cTn assays, applied decision limits for AMI diagnosis, and date of implementation of hs-cTnI assay are listed in Supplementary Table S1. All sites implemented ARCHITECT i2000SR STAT hs-cTnI assay from Abbott, and applied sex-specific thresholds corresponding with a female-specific 99th percentile of 16 ng/L and a male-specific 99th percentile of 34 ng/L. In SWEDEHEART registry only peak troponin levels during admission are registered.

### Diagnosis and in-hospital management

According to clinical primary discharge diagnoses, we categorized patients into cohorts with AMI, unstable angina (UA), heart failure, other cardiac conditions or non-cardiac conditions. In-hospital examinations contained echocardiography and coronary angiography. In-hospital treatment data on intravenous/subcutaneous anticoagulants (unfractionated heparin, low-molecular-weight heparin [LMWH], and fondaparinux), PCI and CABG were collected, and discharge medications included antiplatelets [aspirin, P2Y12 inhibitors], ACEI, ARB, and statins.

### Prognostic evaluation

Information on patient outcome was obtained from mandatory Swedish Patient Registry (hospitalization dates and discharge diagnoses based on International Classification of Diseases, 10th revision, Clinical Modification [ICD-10-CM] codes) and Swedish population registry. Patients were followed for mortality until occurrence of death or May 28th, 2018, and for non-fatal events until December 31st, 2017. Outcomes were MACE and all-cause mortality. MACE was a composite endpoint of all-cause mortality, readmissions for non-fatal AMI (ICD-10-CM I21), heart failure (ICD-10-CM I50) and ischemic stroke (ICD-10-CM I63). During the first 30-days after index hospitalization, it is not possible to separate a new AMI from an index AMI in the Patient Registry. Therefore, only AMI occurring 30 days after index hospitalization were counted.

### Statistical analysis

Differences of clinical characteristics between groups are assessed by Independent Student’s T test or Mann–Whitney-U test, as appropriate. For comparison of proportions, the Chi-squared test is used. The group of interest for management of AMI are patients with troponin levels above diagnostic AMI threshold (Supplementary Table S2). To disentangle the effect on discharge diagnosis, treatment and outcome of lowering diagnostic threshold of AMI to sex-specific 99th percentiles, we classified patients in patients with troponin levels above the respective AMI threshold and patients with troponin levels below the respective AMI threshold per study period. Analysis are conducted for (1) all patients, (2) patients with troponin levels above the respective AMI threshold and (3) patients with troponin levels equal or below (termed “below” throughout the manuscript) the respective AMI threshold, accordingly. Proportion of final diagnosis at discharge and in-hospital treatments are compared between study periods, stratified by sex. Multivariable logistic regression analyses are performed to assess clinical management of hs-cTnI period as compared with cTn period (reference category). The in-hospital examinations and treatments were taken as dependent variable in the model. A minimal sufficient adjustment set was identified using the web-based application DAGitty 3.0^22^ and included admission year only. In addition, adjustment was made for hospital site. Multivariable cox proportional hazard regression analysis were conducted to quantify the relationship with hs-cTnI period (cTn period, reference category) and outcome (MACE, all-cause mortality), adjusting for the same set of covariates as used for logistic regressions. Interaction was tested between sex and hs-cTnI assay, and was considered significant when *P* value < 0.1. Otherwise, statistical significance was reached when *P* value < 0.05. All statistical analysis are performed using SPSS for windows 23.0 (IBM Corp., Armonk, NY, USA).

## Supplementary information

Supplementary Information

## Data Availability

The datasets generated during and/or analyzed during the current study are not publicly available due to ethical restrictions and national laws but are available on reasonable request under the provision that data may not leave the Uppsala University.
