# Supplementary material for: Sex-specific effects of implementing a high-sensitivity troponin I assay in patients with suspected acute coronary syndrome: results from SWEDEHEART registry
Source: Sci Rep. 2020 Sep 17;10:15227. doi: 10.1038/s41598-020-72204-2 (PMC7499170; doi:10.1038/s41598-020-72204-2)
Supplement: Supplementary file 1 — Supplementary Information [file 41598_2020_72204_MOESM1_ESM.docx]

**Online Supplementary Appendix**

**Sex-specific effects of implementing a high-sensitivity troponin I assay in patients with suspected acute coronary syndrome: results from SWEDEHEART registry**

Dorien M Kimenai PhD^1,2^, Bertil Lindahl MD, PhD^3,4^, Tomas Jernberg MD, PhD^5^, Otto Bekers PhD^1,2^, Steven JR Meex PhD^1,2^, Kai M Eggers MD, PhD^3^

^1^Central Diagnostic Laboratory, Maastricht University Medical Center, Maastricht, the Netherlands;

^2^CARIM School for Cardiovascular Diseases, Maastricht University, Maastricht, the Netherlands;

^3^Department of Medical Sciences, Cardiology, Uppsala University, Uppsala, Sweden; ^4^Uppsala Clinical Research Center, Uppsala University, Uppsala, Sweden;

^5^Department of Clinical Sciences, Danderyd University Hospital, Karolinska Institutet, Stockholm, Sweden

Corresponding author:

Dorien Kimenai, PhD

Central Diagnostic Laboratory

Maastricht University Medical Center

Post office box 5800

6202 AZ Maastricht

Tel: +31 (0)43 3871399

Fax: +31 (0)43 3874692

Email: Dorien.Kimenai@ed.ac.uk

**Supplemental Table 1. Study population and cardiac troponin information per each hospital**

|  | **cTn period** | | | **hs-cTnI period*** | **Date of method change** | **Start of observation period** |
| --- | --- | --- | --- | --- | --- | --- |
| **Hospital** | **n (%)** | **Type of cTn assay** | **AMI threshold** | **n (%)** |  |  |
| Enköping | 138 (1.1%) | cTnI (Stratus CS) | 0.07 mg/L | 134 (1.1%) | 02-01-2017 | 18-07-2015 |
| Falun | 2233 (17.9%) | cTnI (Abbott) | 0.10 mg/L | 2130 (17.1%) | 26-01-2015 | 16-04-2013 |
| Karlskoga | 563 (4.5%) | cTnI (Abbott) | 0.07 mg/L | 548 (4.4%) | 11-11-2014 | 30-07-2012 |
| Köping | 205 (1.6%) | cTnI (Stratus CS) | 0.07 mg/L | 204 (1.6%) | 17-05-2017 | 26-08-2016 |
| Lindesberg | 582 (4.7%) | cTnI (Abbott) | 0.07 mg/L | 575 (4.6%) | 11-11-2014 | 05-01-2012 |
| Mora | 704 (5.6%) | cTnI (Stratus CS) | 0.10 mg/L | 708 (5.7%) | 04-02-2015 | 25-11-2012 |
| Visby | 799 (6.4%) | cTnI (Abbott) | 0.03 mg/L | 705 (5.6%) | 07-04-2014 | 16-11-2011 |
| Västeras | 257 (2.1%) | cTnI (Stratus CS) | 0.07 mg/L | 242 (1.9%) | 17-05-2017 | 19-07-2016 |
| Örebro | 904 (7.2%) | cTnI (Abbott) | Before 3/11/2010, 0.10 mg/L  Since 3/11/2010, 0.07 mg/L | 858 (6.9%) | 11-11-2014 | 03-08-2010 |

Abbreviations: AMI, acute myocardial infarction; cTn, conventional cardiac troponin; hs-cTnI, high-sensitivity cardiac troponin I. ^*^ hs-cTnI assay (Abbott), female-specific threshold 16 ng/L; male-specific threshold 34 ng/L.

**Supplemental Table 2. Baseline characteristics and in-hospital treatment of patients with troponin levels above the respective AMI threshold per study period, stratified by sex**

|  | **All** | | | **Women** | | | **Men** | | | |
| --- | --- | --- | --- | --- | --- | --- | --- | --- | --- | --- |
|  | cTn period  (n =3497) | hs-cTnI period  (n =4476) | P-value | cTn period  (n =1309) | hs-cTnI period  (n =1823) | P-value | cTn period  (n =2188) | hs-cTnI period  (n = 2653) | P-value |  |
| Age (years) | 74 (65-83) | 74 (66-82) | 0.710 | 77 (67-85) | 76 (69-84) | 0.512 | 72 (64-81) | 72 (64-80) | 0.452 |  |
| Female sex | 1309 (37.4%) | 1823 (40.7%) | 0.003 | - | - | - | - | - | - |  |
| **Risk factors** |  |  |  |  |  |  |  |  |  |  |
| Current smoking | 502 (14.4%) | 619 (13.8%) | 0.516 | 182 (13.9%) | 257 (14.1%) | 0.917 | 320 (14.6%) | 362 (13.6%) | 0.340 |  |
| Hypertension | 1844 (52.7%) | 2817 (63.0%) | <0.001 | 781 (59.7%) | 1223 (67.1%) | <0.001 | 1063 (48.6%) | 1594 (60.1%) | <0.001 |  |
| Diabetes | 958 (27.4%) | 1271 (28.4%) | 0.315 | 365 (27.9%) | 520 (28.5%) | 0.717 | 593 (27.1%) | 751 (28.3%) | 0.350 |  |
| Hyperlipidemia | 1434 (41.0%) | 1955 (43.7%) | 0.017 | 489 (37.4%) | 738 (40.5%) | 0.081 | 945 (43.2%) | 1217 (45.9%) | 0.059 |  |
| Body mass index (kg/m^2^) | 27 (24-30) | 27 (24-30) | 0.779 | 27 (23-30) | 27 (24-31) | 0.255 | 27 (25-30) | 27 (24-30) | 0.626 |  |
| eGFR (CKD-EPI, mL/kg/m^2^) | 68 (47-86) | 73 (52-88) | <0.001 | 63 (42-82) | 67 (48-84) | <0.001 | 71 (50-88) | 76 (56-89) | <0.001 |  |
| **History** |  |  |  |  |  |  |  |  |  |  |
| Previous AMI | 1374 (39.3%) | 1646 (36.8%) | 0.023 | 479 (36.6%) | 629 (34.5%) | 0.240 | 895 (40.9%) | 1017 (38.3%) | 0.072 |  |
| Previous PCI/ CABG | 1105 (31.6%) | 1398 (31.3%) | 0.752 | 330 (25.2%) | 461 (25.3%) | 0.967 | 775 (35.4%) | 937 (35.4%) | 0.976 |  |
| Heart failure | 611 (17.5%) | 637 (14.2%) | <0.001 | 225 (17.2%) | 228 (12.5%) | <0.001 | 386 (17.6%) | 409 (15.4%) | 0.043 |  |
| Previous stroke |  |  |  |  |  |  |  |  |  |  |
| COPD | 289 (8.3%) | 370 (8.3%) | 1.000 | 121 (9.2%) | 184 (10.1%) | 0.464 | 168 (7.7%) | 186 (7.0%) | 0.376 |  |
| Dementia | 24 (0.7%) | 9 (0.2%) | 0.001 | 10 (0.8%) | 5 (0.3%) | 0.065 | 14 (0.6%) | 4 (0.2%) | 0.007 |  |
| Previous/present cancer | 200 (5.7%) | 178 (4.0%) | <0.001 | 52 (4.0%) | 56 (3.1%) | 0.197 | 148 (6.8%) | 122 (4.6%) | 0.001 |  |
| **ECG findings** |  |  |  |  |  |  |  |  |  |  |
| Sinus rhythm | 2782 (80.7%) | 3663 (82.1%) | 0.096 | 1036 (80.2%) | 1506 (82.8%) | 0.066 | 1746 (80.9%) | 2157 (81.7%) | 0.503 |  |
| Atrial fibrillation/ flutter | 550 (15.9%) | 633 (14.2%) | 0.031 | 223 (17.3%) | 255 (14.0%) | 0.015 | 357 (15.2%) | 378 (14.3%) | 0.413 |  |
| ST-segment depression | 985 (28.6%) | 1251 (28.1%) | 0.632 | 402 (31.1%) | 513 (28.2%) | 0.079 | 583 (27.0%) | 738 (27.9%) | 0.475 |  |
| T-wave inversion | 398 (11.5%) | 440 (9.9%) | 0.018 | 179 (13.9%) | 191 (10.5%) | 0.005 | 219 (10.1%) | 249 (9.4%) | 0.406 |  |
| Other ST segment changes | 474 (21.7%) | 963 (21.6%) | 0.956 | 276 (21.4%) | 387 (21.3%) | 0.965 | 471 (21.8%) | 576 (21.8%) | 1.000 |  |
| No ST-segment changes | 1319 (38.2%) | 1805 (40.5%) | 0.044 | 434 (33.6%) | 727 (40.0%) | <0.001 | 885 (41.0%) | 1078 (40.8%) | 0.906 |  |
| **In-hospital examinations and interventions** | | | | | | | | | |  |
| Echocardiography | 1962 (56.1%) | 2809 (62.8%) | <0.001 | 689 (52.6%) | 1001 (60.4%) | <0.001 | 1273 (58.2%) | 1708 (64.4%) | <0.001 |  |
| Coronary angiography | 2123 (60.7%) | 3059 (68.3%) | <0.001 | 676 (51.6%) | 1112 (61.0%) | <0.001 | 1447 (66.1%) | 1947 (73.4%) | <0.001 |  |
| PCI | 1281 (36.6%) | 1739 (38.9%) | 0.043 | 350 (26.7%) | 508 (27.9%) | 0.490 | 931 (42.6%) | 1231 (46.4%) | 0.008 |  |
| CABG | 68 (2.0%) | 119 (2.7%) | 0.073 | 15 (1.2%) | 26 (1.4%) | 0.633 | 53 (2.5%) | 93 (3.5%) | 0.052 |  |
| **Coronary status*** | | | | | | | | | |  |
| Non-conclusive | 7 (0.3%) | 0 | 0.002 | 4 (0.6%) | 0 | 0.021 | 3 (0.2%) | 0 | 0.077 |  |
| Normal/ atheromatosis | 456 (21.9%) | 811 (27.1%) | <0.001 | 216 (31.6%) | 448 (40.5%) | <0.001 | 240 (17.1%) | 363 (19.2%) | 0.133 |  |
| 1-2 vessel disease | 1207 (57.9%) | 1573 (52.5%) | <0.001 | 356 (52.1%) | 501 (45.3%) | 0.006 | 851 (60.7%) | 1072 (56.7%) | 0.024 |  |
| 3 vessel disease/ left main | 416 (19.9%) | 612 (20.4%) | 0.696 | 107 (15.7%) | 156 (14.1%) | 0.372 | 309 (22.0%) | 456 (24.1%) | 0.169 |  |
| **Left ventricular ejection fraction †** | | | | | | | | | |  |
| ≥ 50% | 1164 (60.0%) | 1855 (66.5%) | <0.001 | 427 (62.7%) | 741 (67.9%) | 0.027 | 737 (58.5%) | 1114 (65.5%) | <0.001 |  |
| 31 – 49% | 580 (29.9%) | 711 (25.5%) | 0.001 | 193 (28.3%) | 265 (24.3%) | 0.066 | 387 (30.7%) | 446 (26.2%) | 0.007 |  |
| ≤ 30%% | 196 (10.1%) | 225 (8.1%) | 0.017 | 61 (9.0%) | 85 (7.8%) | 0.424 | 135 (10.7%) | 140 (8.2%) | 0.025 |  |
| Duration of hospital stays (days) | 4 (2-5) | 3 (2-5) | <0.001 | 4 (2-6) | 3 (2-5) | 0.512 | 3 (2-5) | 3 (2-5) | 0.452 |  |
| **Medication at discharge ‡** | | | | | | | | | |  |
| Aspririn | 2732 (81.3%) | 3240 (75.4%) | <0.001 | 971 (76.9%) | 1252 (71.5%) | 0.001 | 1761 (84.0%) | 1988 (78.2%) | <0.001 |  |
| P2Y12 inhibitors | 2193 (65.3%) | 2807 (65.4%) | 0.961 | 731 (57.9%) | 1007 (57.5%) | 0.823 | 1462 (69.7%) | 1800 (70.8%) | 0.439 |  |
| Anticoagulants | 432 (12.9%) | 437 (10.2%) | <0.001 | 167 (13.2%) | 172 (9.8%) | 0.004 | 265 (12.6%) | 265 (10.4%) | 0.020 |  |
| Β-blockers | 2719 (80.9%) | 3435 (80.0%) | 0.296 | 1017 (80.6%) | 1383 (78.9%) | 0.272 | 1702 (81.2%) | 2052 (80.7%) | 0.708 |  |
| ACEI/ARB | 2366 (70.4%) | 3179 (74.0%) | 0.001 | 840 (66.6%) | 1280 (73.1%) | <0.001 | 1526 (72.8%) | 1899 (74.7%) | 0.149 |  |
| Statins | 2592 (77.2%) | 3417 (79.6%) | 0.012 | 892 (70.7%) | 1276 (72.8%) | 0.203 | 1700 (81.1%) | 2141 (84.2%) | 0.006 |  |

Abbreviations: ACEI, angiotensin-converting-enzyme inhibitor; AMI, acute myocardial infarction; ARB, Angiotensin II receptor blockers; CABG, coronary artery bypass graft; CKD-EPI, Chronic Kidney Disease Epidemiology Collaboration equation; COPD, chronic obstructive pulmonary disease; eGFR, estimated glomerular filtration rate; PCI, percutaneous coronary intervention. *n=5082. †n=4731. ‡assessed in in-hospital survivors (n=7973).

**Supplemental Table 3. Baseline characteristics and in-hospital treatment of patients with troponin levels below the respective AMI threshold per study period, stratified by sex**

|  | **All** | | | **Women** | | | **Men** | | | |
| --- | --- | --- | --- | --- | --- | --- | --- | --- | --- | --- |
|  | cTn period  (n =2888) | hs-cTnI period  (n =1628) | P-value | cTn period  (n =1158) | hs-cTnI period  (n =529) | P-value | cTn period  (n =1730) | hs-cTnI period  (n =1099 ) | P-value |  |
| Age (years) | 68 (58-77) | 68 (59-75) | 0.316 | 70 (61-79) | 69 (59-78) | 0.135 | 67 (57-75) | 67 (59-74) | 0.540 |  |
| Female sex | 1158 (40.1%) | 529 (32.5%) | <0.001 | - | - | - | - | - | - |  |
| **Risk factors** |  |  |  |  |  |  |  |  |  |  |
| Current smoking | 365 (12.7%) | 181 (11.1%) | 0.128 | 111 (9.6%) | 57 (529%) | 0.484 | 254 (14.8%) | 124 (11.3%) | 0.009 |  |
| Hypertension | 1343 (46.6%) | 969 (59.5%) | <0.001 | 560 (48.4%) | 318 (60.1%) | <0.001 | 783 (45.3%) | 651 (59.2%) | <0.001 |  |
| Diabetes | 586 (20.3%) | 384 (23.6%) | 0.011 | 217 (18.8%) | 103 (19.5%) | 0.738 | 369 (21.4%) | 281 (25.6%) | 0.010 |  |
| Hyperlipidemia | 1284 (44.5%) | 845 (51.9%) | <0.001 | 427 (36.9%) | 236 (44.7%) | 0.003 | 857 (49.6%) | 609 (55.4%) | 0.003 |  |
| Body mass index (kg/m^2^) | 27 (24-30) | 27 (25-30) | 0.075 | 27 (24-30) | 27 (24-30) | 0.527 | 27 (25-30) | 27 (25-30) | 0.131 |  |
| eGFR (CKD-EPI, mL/kg/m^2^) | 79 (61-92) | 84 (68-94) | <0.001 | 77 (58-90) | 82 (66-93) | <0.001 | 81 (63-93) | 85 (71-94) | <0.001 |  |
| **History** |  |  |  |  |  |  |  |  |  |  |
| Previous AMI | 1011 (35.0%) | 587 (36.1%) | 0.496 | 332 (28.7%) | 147 (27.8%) | 0.727 | 67.9 (39.3%) | 440 (40.0%) | 0.693 |  |
| Previous PCI/ CABG | 944 (32.8%) | 632 (38.8%) | <0.001 | 258 (22.3%) | 134 (25.4%) | 0.172 | 686 (39.7%) | 498 (45.3%) | 0.003 |  |
| Heart failure | 323 (11.2%) | 140 (8.6%) | 0.006 | 101 (8.7%) | 22 (4.2%) | 0.001 | 222 (12.9%) | 118 (10.7%) | 0.097 |  |
| Previous stroke | 248 (8.6%) | 126 (7.7%) | 0.339 | 87 (7.5%) | 37 (7.0%) | 0.763 | 161 (9.3%) | 89 (8.1%) | 0.278 |  |
| COPD | 204 (7.1%) | 90 (5.5%) | 0.045 | 85 (7.3%) | 38 (7.2%) | 1.000 | 119 (6.9%) | 52 (4.7%) | 0.019 |  |
| Dementia | 8 (0.3%) | 3 (0.2%) | 0.756 | 0 | 0 | - | 8 (0.3%) | 3 (0.2%) | 0.545 |  |
| Previous/present cancer | 112 (3.9%) | 60 (3.7%) | 0.808 | 24 (2.1%) | 14 (2.6%) | 0.481 | 88 (5.1%) | 46 (4.2%) | 0.318 |  |
| **ECG findings** |  |  |  |  |  |  |  |  |  |  |
| Sinus rhythm | 2371 (85.4%) | 1439 (88.6%) | 0.003 | 975 (87.9%) | 476 (90.3%) | 0.157 | 1396 (83.8%) | 963 (87.7%) | 0.004 |  |
| Atrial fibrillation/ flutter | 331 (11.9%) | 152 (9.4%) | 0.008 | 115 (10.4%) | 47 (8.9%) | 0.377 | 216 (13.0%) | 105 (9.6%) | 0.006 |  |
| ST-segment depression | 242 (8.7%) | 162 (10.0%) | 0.176 | 119 (10.7%) | 59 (11.2%) | 0.799 | 123 (7.4%) | 103 (9.4%) | 0.065 |  |
| T-wave inversion | 218 (7.9%) | 123 (7.6%) | 0.770 | 93 (8.4%) | 39 (7.4%) | 0.560 | 125 (7.5%) | 84 (7.7%) | 0.883 |  |
| Other ST segment changes | 434 (15.7%) | 248 (15.3%) | 0.763 | 135 (12.2%) | 57 (10.8%) | 0.460 | 299 (18.0%) | 191 (17.4%) | 0.722 |  |
| No ST-segment changes | 1878 (67.7%) | 1092 (67.2%) | 0.714 | 760 (68.7%) | 372 (70.6%) | 0.465 | 1118 (67.1%) | 720 (65.6%) | 0.410 |  |
| **In-hospital examinations and interventions** | | | | | | | | | |  |
| Echocardiography | 597 (20.7%) | 527 (32.4%) | <0.001 | 212 (18.3%) | 169 (31.9%) | <0.001 | 385 (22.3%) | 358 (32.6%) | <0.001 |  |
| Coronary angiography | 763 (26.4%) | 757 (46.5%) | <0.001 | 269 (23.2%) | 199 (37.6%) | <0.001 | 494 (28.6%) | 558 (50.8%) | <0.001 |  |
| PCI | 261 (9.0%) | 292 (17.9%) | <0.001 | 82 (7.1%) | 49 (9.3%) | 0.141 | 179 (10.3%) | 243 (22.1%) | <0.001 |  |
| CABG | 10 (0.4%) | 11 (0.7%) | 0.260 | 3 (0.3%) | 1 (0.2%) | 1.000 | 7 (0.5%) | 10 (0.9%) | 0.216 |  |
| **Coronary status*** | | | | | | | | | |  |
| Non-conclusive | 1 (0.1%) | 1 (0.1%) | 1.000 | 0 | 0 | - | 1 (0.2%) | 1 (0.2%) | 1.000 |  |
| Normal/ atheromatosis | 369 (46.2%) | 362 (47.9%) | 0.542 | 152 (54.3%) | 123 (61.8%) | 0.111 | 217 (41.9%) | 239 (42.9%) | 0.758 |  |
| 1-2 vessel disease | 324 (40.6%) | 314 (41.5%) | 0.718 | 102 (36.4%) | 64 (32.2%) | 0.381 | 222 (42.9%) | 250 (44.9%) | 0.539 |  |
| 3 vessel disease/ left main | 104 (13.0%) | 79 (10.4%) | 0.116 | 26 (9.3%) | 12 (6.0%) | 0.231 | 78 (15.1%) | 67 (12.0%) | 0.154 |  |
| **Left ventricular ejection fraction †** | | | | | | | | | |  |
| ≥ 50% | 466 (79.4%) | 433 (82.8%) | 0.168 | 178 (86.0%) | 149 (88.7%) | 0.535 | 288 (75.8%) | 284 (80.0%) | 0.183 |  |
| 31 – 49% | 84 (14.3%) | 69 (13.2%) | 0.602 | 23 (11.1%) | 17 (10.1%) | 0.867 | 61 (16.1%) | 52 (14.6%) | 0.610 |  |
| ≤ 30%% | 37 (6.3%) | 21 (4.0%) | 0.105 | 6 (2.9%) | 2 (1.2%) | 0.305 | 31 (8.2%) | 19 (5.4%) | 0.144 |  |
| Duration of hospital stays (days) | 1 (1-2) | 1 (1-3) | <0.001 | 1 (1-2) | 1 (1-2) | 0.135 | 1 (1-2) | 1 (1-3) | 0.540 |  |
| **Medication at discharge ‡** | | | | | | | | | |  |
| Aspririn | 1426 (52.1%) | 848 (52.5%) | 0.777 | 484 (44.0%) | 226 (43.2%) | 0.789 | 942 (57.5%) | 622 (57.0%) | 0.813 |  |
| P2Y12 inhibitors | 589 (21.5%) | 470 (29.1%) | <0.001 | 188 (17.1%) | 102 (19.5%) | 0.267 | 401 (24.5%) | 368 (33.7%) | <0.001 |  |
| Anticoagulants | 375 (13.7%) | 124 (7.7%) | <0.001 | 135 (12.3%) | 30 (5.7%) | <0.001 | 240 (14.7%) | 94 (8.6%) | <0.001 |  |
| Β-blockers | 1584 (57.9%) | 939 (58.2%) | 0.849 | 597 (54.3%) | 275 (52.6%) | 0.523 | 987 (60.3%) | 664 (60.9%) | 0.780 |  |
| ACEI/ARB | 1374 (50.2%) | 980 (60.7%) | <0.001 | 503 (45.7%) | 294 (56.2%) | <0.001 | 871 (53.2%) | 686 (62.9%) | <0.001 |  |
| Statins | 1537 (56.1%) | 1053 (65.2%) | <0.001 | 528 (48.0%) | 294 (56.2%) | 0.002 | 1009 (61.6%) | 759 (69.6%) | <0.001 |  |

Abbreviations: ACEI, angiotensin-converting-enzyme inhibitor; AMI, acute myocardial infarction; ARB, Angiotensin II receptor blockers; CABG, coronary artery bypass graft; CKD-EPI, Chronic Kidney Disease Epidemiology Collaboration equation; COPD, chronic obstructive pulmonary disease; eGFR, estimated glomerular filtration rate; PCI, percutaneous coronary intervention. *n=1554. †n=1110. ‡assessed in in-hospital survivors (n=4516).

**Supplemental Table 4. Risk on major adverse cardiac events and all-cause mortality for hs-cTnI period in patients with troponin levels above and below the respective AMI threshold**

|  | **Patients with troponin levels above the respective AMI threshold** | | | | **Patients with troponin levels below the respective AMI threshold** | | | |
| --- | --- | --- | --- | --- | --- | --- | --- | --- |
|  | **N** | **HR (95% CI)** | **P-value** | **P_int_** | **N** | **HR (95% CI)** | **P-value** | **P_int_** |
| **All** |  |  |  |  |  |  |  |  |
| MACE | 6196 | 0.90 (0.78-1.03) | 0.114 | 0.576 | 3539 | 0.67 (0.48-0.94) | 0.021 | 0.824 |
| All-cause mortality | 6631 | 0.87 (0.74-1.02) | 0.076 | 0.210 | 3655 | 0.56 (0.37-0.55) | 0.007 | 0.170 |
| **Women** |  |  |  |  |  |  |  |  |
| MACE | 2403 | 0.85 (0.69-1.05) | 0.128 | - | 1382 | 0.79 (0.45-1.40) | 0.425 | - |
| All-cause mortality | 2555 | 0.88 (0.69-1.12) | 0.290 | - | 1422 | 0.73 (0.38-1.41) | 0.348 | - |
| **Men** |  |  |  |  |  |  |  |  |
| MACE | 3793 | 0.91 (0.76-1.09) | 0.318 | - | 2157 | 0.61 (0.40-0.92) | 0.018 | - |
| All-cause mortality | 4076 | 0.84 (0.68-1.04) | 0.106 | - | 2233 | 0.47 (0.27-0.82) | 0.007 | - |

Abbreviations: hs-cTnI, high-sensitivity cardiac troponin I; MACE, major adverse cardiovascular events. Reference category; troponin positive group, troponin-positive patients from cTn period; troponin-negative group, troponin-negative patients from cTn period. Model adjusted for sex (if appropriate), year of admission and hospital site. P_interaction_ refers to the interaction term of the used cTn assay (conventional or high-sensitivity) on the association of sex with the respective examination or treatment (cTn assay*sex).

**
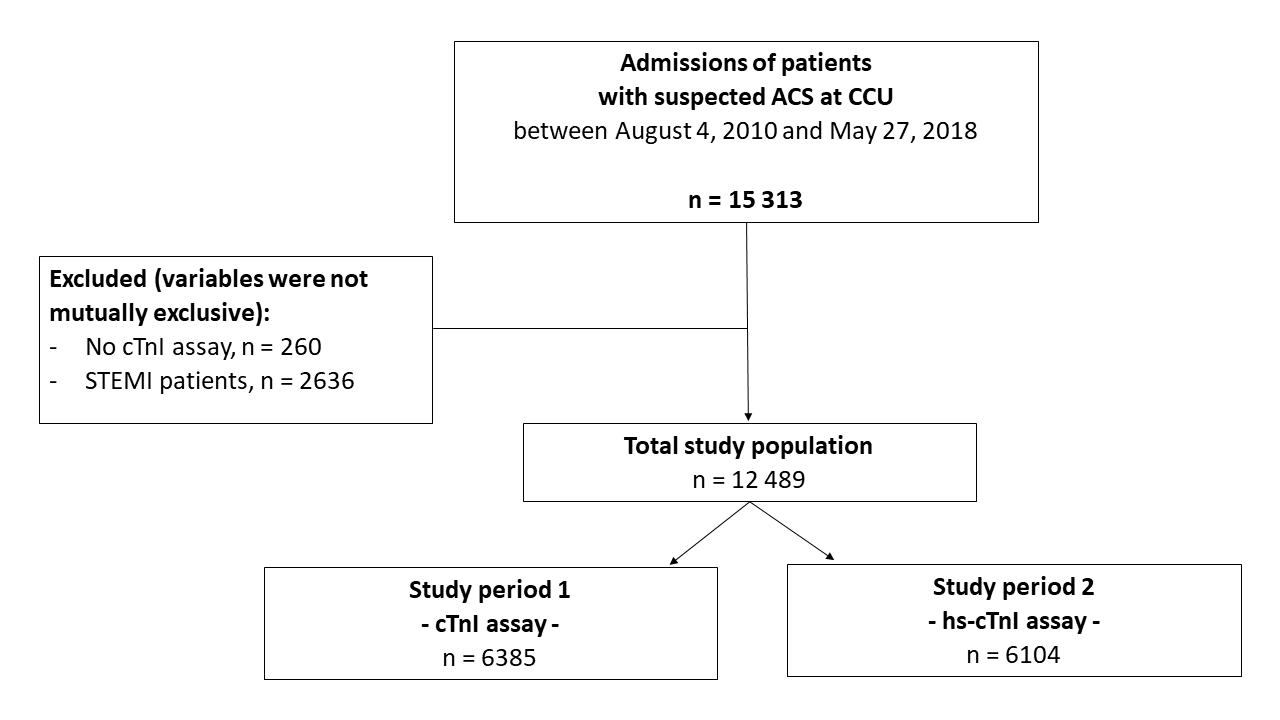
**

**Supplemental Figure 1.** Study flow diagram. Abbreviations: ACS, acute coronary syndrome; CCU, coronary care unit; cTnI, cardiac troponin I; hs-cTnI, high-sensitivity cardiac troponin I; STEMI, ST-segment elevation myocardial infarction.
